# Effects of a Community-Based Multi-Component Intervention on Subjective Well-Being in Older Adults: The Chofu–Digital–Choju Project in Japan

**DOI:** 10.3390/geriatrics11020027

**Published:** 2026-03-03

**Authors:** Tsubasa Nakada, Kayo Kurotani, Satoshi Seino, Takako Kozawa, Shinichi Murota, Miki Eto, Junko Shimasawa, Yumiko Shimizu, Shinobu Tsurugano, Fuminori Katsukawa, Kazunori Sakamoto, Hironori Washizaki, Yo Ishigaki, Maki Sakamoto, Keiki Takadama, Keiji Yanai, Osamu Matsuo, Chiyoko Kameue, Hitomi Suzuki, Kazunori Ohkawara

**Affiliations:** 1 Graduate School of Informatics and Engineering, The University of Electro-Communications, Tokyo 182-8585, Japan; tsubasanakada@uec.ac.jp (T.N.); maki.sakamoto@uec.ac.jp (M.S.); yanai@cs.uec.ac.jp (K.Y.); ma004017@edu.cc.uec.ac.jp (O.M.); kameue.chiyoko@uec.ac.jp (C.K.); 2 Graduate School of Life Sciences, Showa Women’s University, Tokyo 154-8533, Japan; k-kurotani@swu.ac.jp; 3 Institute of Well-Being, Yamagata University, Yamagata 990-9585, Japan; seino.s@med.id.yamagata-u.ac.jp; 4 Faculty of Human Health, Komazawa Women’s University, Tokyo 206-8511, Japan; t-kozawa@komajo.ac.jp; 5 Faculty of Humanities and Social Sciences, Tokyo Metropolitan University, Tokyo 192-0397, Japan; shin1@tmu.ac.jp; 6 Faculty of Human Sciences, Osaka University of Economics, Osaka 533-8533, Japan; eto@osaka-ue.ac.jp; 7 School of Nursing, The Jikei University, Tokyo 182-8570, Japan; jshimasawa@jikei.ac.jp (J.S.); yumiko_shimizu@jikei.ac.jp (Y.S.); 8 Center for Health Sciences and Counseling, Kyushu University, Fukuoka 819-0395, Japan; tsurugano@chc.kyushu-u.ac.jp; 9 Sports Medicine Research Center, Keio University, Yokohama 223-8521, Japan; fuminori@keio.jp; 10 Green Computing Systems Research Organization, Waseda University, Tokyo 169-8050, Japan; k.sakamoto@aoni.waseda.jp; 11 Faculty of Science and Engineering, School of Fundamental Science and Engineering, Waseda University, Tokyo 169-8050, Japan; washizaki@waseda.jp; 12 Research Center for Realizing Sustainable Societies, The University of Electro-Communications, Tokyo 182-8585, Japan; ishigaki@uec.ac.jp; 13 Information Technology Center, The University of Tokyo, Chiba 277-0882, Japan; takadama@g.ecc.u-tokyo.ac.jp; 14 Office for Research Management, The University of Electro-Communications, Tokyo 182-8585, Japan; suzuki.hitomi@uec.ac.jp

**Keywords:** information and communication technology, health literacy, social connection, Internet use

## Abstract

**Background:** Subjective well-being (SWB) is an essential indicator of successful aging. Although social connections enhance SWB among older adults, few interventions have integrated community-based approaches with information and communication technology (ICT). This study evaluated the Chofu–Digital–Choju (CDC) project, a multi-component community intervention fostering in-person and online social connections among community-dwelling older adults in urban Japan. **Methods:** This quasi-experimental study (January 2022 to March 2024) included community-dwelling older adults aged 65–84 years in Chofu City, Tokyo, Japan. The intervention consisted of online classes, community hubs as local third places, and community events. Baseline and follow-up data were collected using self-administered questionnaires. Propensity score matching (1:1) was used to reduce selection bias, and generalized estimating equations were applied to evaluate the intervention effects. The primary outcome was SWB (Cantril Ladder). The secondary outcomes included social isolation, neighborhood relationships, social participation, health literacy, psychological health, physical activity, and ICT use. **Results:** Among the 1599 participants who completed both surveys, 209 (13.1%) participated in at least one CDC intervention component. After propensity score matching, 195 pairs were analyzed. No significant interaction effect was observed for SWB (β = 0.08, 95% confidence interval [CI]: −0.20, 0.37; *p* = 0.565). However, a significant interaction effect favored the intervention group for Internet usage frequency (odds ratio = 1.53, 95% CI: 1.08, 2.16; *p* = 0.016). A significant borderline interaction was also observed in health literacy (β = 0.13, 95% CI: −0.00, 0.26; *p* = 0.056), which reached significance in covariate-adjusted sensitivity analysis (*p* = 0.044). Subgroup analyses revealed that community hub participants showed significant interaction effects in health literacy (*p* = 0.021) and a trend toward reduced depressive symptoms (*p* = 0.084). **Conclusions:** The CDC intervention did not improve SWB over 2 years but enhanced Internet use and supported health literacy and depressive symptoms, particularly among hub participants. Community-based, multi-component interventions that integrate online and in-person activities may foster digital inclusion and specific health behaviors. Although SWB did not change in this study, these proximal gains may serve as foundational steps for long-term improvement. The study protocol was preregistered in the UMIN Clinical Trials Registry (UMIN000051393; Registered on 21 June 2023).

## 1. Introduction

Japan is a super-aged nation, with projections indicating that nearly one-third of its population will be aged 65 or older by 2025 [1]. The aging process is often accompanied by challenges such as declining self-rated health, cognitive impairment, and impactful life events like bereavement, all of which can negatively affect well-being [2,3,4]. Consequently, promoting subjective well-being (SWB) among older adults has become a key public health priority for successful aging. SWB is defined as the state in which individuals realize their abilities, cope with normal life stresses, function productively, contribute to their community, and find contentment [5]. Maintaining high levels of SWB is essential for successful aging [6]. Notably, older adults with high levels of SWB demonstrate low risks of frailty and mortality [7,8,9]. A meta-analysis of longitudinal studies revealed an effect size of 0.18 for health outcomes, demonstrating the difference in standard deviation (SD) between individuals with low and high SWB [10].

Social networks are recognized as an important determinant of high SWB. Psychological intervention programs involving social interactions positively affect SWB [11]. Social relationships have consistently been identified as predictors of well-being [12]. A previous review identified the following four key strategies for enhancing social connections among older adults: multigenerational programs, aging-friendly communities, group-based physical activity approaches, and the use of information and communication technology (ICT) [13]. Of these, ICT use warrants particular attention because of its indirect influence on well-being through enhancing social capital [14], which encompasses networks, norms, and social trust that facilitate coordination and cooperation for mutual benefit. A comprehensive review has established that Internet use among older adults is related to improved quality of life (QoL), with most studies demonstrating benefits, such as communicating with family and friends, maintaining broader social networks, accessing information, and participating in online leisure activities [15]. The importance of social connections is particularly pronounced in Japan, a nation at the forefront of global aging. Domestic longitudinal research has identified social isolation, especially a lack of interaction with friends and social participation, as a key risk factor for a wide range of adverse health and well-being outcomes [9,16]. In response, promoting in-person participation in community gatherings has become a local strategy to prevent functional disability and enhance well-being [17].

Recent systematic reviews have revealed a nuanced landscape for interventions targeting loneliness, highlighting the importance of the delivery format. Technology-based interventions are effective in maintaining existing social ties; however, their impact can be limited by barriers, such as digital literacy and usability [18,19]. Conversely, non-technology-based in-person interventions often yield larger effect sizes, underscoring the irreplaceable value of face-to-face social interactions [20]. This evidence suggests the need for a synergistic, hybrid approach. A recent network meta-analysis that directly compared various formats concluded that combining face-to-face and online methods was the most effective approach for reducing loneliness [21]. Such an integrated model is hypothesized to be more robust, as the in-person component can directly address and overcome barriers that often limit the success of technology-only initiatives.

While previous community-based interventions aimed at improving the SWB of older adults have been implemented [22], to the best of our knowledge, existing studies have often examined in-person or digital interventions in isolation. Few studies have evaluated a “hybrid” model that integrates both formats in a real-world community setting. To address this gap, this study aimed to examine the Chofu–Digital–Choju (CDC; “Choju” meaning longevity in Japanese) project, a community-based multi-component intervention launched in 2022 in Chofu City, Tokyo, Japan. We evaluated the effects of this community-based intervention designed to foster both in-person and online social connections on SWB and related social interaction indicators, including social isolation, neighborhood relationships, and social participation among community-dwelling older adults.

## 2. Methods

### 2.1. Study Design

This paper reports the findings from a 2-year follow-up of the Chofu–Digital–Choju (CDC) project, a community-based, quasi-experimental study [23] designed to enhance SWB in older adults. The detailed study protocol, participant recruitment methods, and baseline characteristics have been published previously [24]. In brief, the CDC project was conducted from January 2022 to March 2024, in collaboration with industry, academia, and the government. The project aimed to foster in-person and online social connections to enhance the SWB of older adults. Baseline and follow-up data were collected in January 2022 and October 2023, respectively, via self-administered questionnaires. The Ethics Committee of our institution approved the study protocol (approval on 16 December 2021; 21068). The trial was registered in the UMIN Clinical Trials Registry (UMIN000051393). This study was reported in accordance with the Transparent Reporting of Evaluations with Nonrandomized Designs (TREND) statement (Table S1) [25].

### 2.2. Study Setting and Participants

The target districts for this study were selected through consultation with city and social welfare council employees based on previous surveys and demographic trends. Chofu City, located in the middle of Tokyo, has a population of 238,311 (115,964 men and 122,347 women) as of 1 October 2021, with 51,536 individuals (22,018 men and 29,518 women) aged ≥65 years, constituting 21.6% of older adults [26]. These districts are located in the south and north of the city, where more than half of the households reside in apartment buildings and detached houses, respectively [27].

The sample size was determined through several steps, as follows: first, a previous review by Bolier et al. indicated that positive psychology interventions showed an effect size of 0.34 for SWB [28]. To detect this effect using a *t*-test, 137 individuals were required in both the intervention and control groups (with a statistical power and risk level of 0.8 and 0.05, respectively). Second, based on a previous survey [29], we estimated a dropout rate of approximately 20% over two years. Third, the expected valid response rate at baseline and follow-up was approximately 80%, based on previous baseline surveys [30,31]. The percentage of participants required in the intervention group was approximately 10% of the total participants. From these estimates, we calculated a baseline survey sample size of 2675 participants.

The study population comprised 3742 community-dwelling individuals who were eligible participants aged 65–84 years as of 1 October 2022, living independently in the two districts of Chofu City, Tokyo, Japan. Individuals aged >85 years were excluded, as the response rate for employment was extremely low according to a previous study [31]. Informed consent was obtained through a questionnaire administered by the municipal government. While the municipality maintained the correspondence table linking personal identifiers to the study data, researchers received only anonymized data after participants had the opportunity to opt out.

### 2.3. Intervention

Following the baseline survey, participants were offered a multi-component intervention designed to foster social connections. The full intervention protocol is detailed in our baseline paper [24]. The key components as implemented were:

Online Classes: We conducted structured online group sessions that integrated exercise, nutrition education, and cognitive training. The program consisted of 75 min weekly sessions over a six-week period. To foster communication, the classes incorporated facilitated, photo-sharing-based conversations aimed at enhancing interpersonal connections [32]. The exercise component focused on fundamental movements essential for daily activities and was adapted for a home-based online format. A total of 23 groups successfully completed this program during the intervention period.

Community Hub: A physical “third place” was established in the community, operating three times per week with volunteer support. This hub provided a space for informal social interaction, where volunteers offered emotional support through empathetic listening [33,34]. To encourage regular visits, the hub also provided practical support, including consultations on smartphone usage and access to various health-measuring devices (e.g., blood pressure, vegetable intake, and cognitive assessment tools).

Community Events: A series of community-based events were organized, targeting a wide range of age groups. During the study, a total of 47 sessions were held. These included experiential health sessions focused on exercise and health assessments (17 sessions) and targeted smartphone classes to improve digital literacy (30 sessions).

### 2.4. Measurements

We collected data on the primary outcome, SWB, and several secondary outcomes using a self-administered questionnaire. The secondary outcomes encompassed four domains: psychosocial function, physical activity and function, dietary habits, and ICT use. We also gathered information on demographics, socioeconomic status, and health and lifestyle factors. To ensure validity and reliability, the questionnaire was constructed primarily using the following validated scales established in previous studies. Additionally, the questions were developed under the supervision of experts in community health and program evaluation. The self-administered questionnaire, mirroring the items from the baseline survey, was distributed to the participants, excluding those who died or relocated from the study area during the 2-year follow-up survey.

### 2.5. Primary Outcome Measure

Our primary outcome, SWB, was assessed using the Cantril Ladder, which evaluates various aspects of people’s attitudes toward their lives and components in various aspects [35]. This instrument asks respondents to rate their current life on an 11-point scale, where 0 represents the worst possible life and 10 represents the best possible life.

### 2.6. Secondary Outcome Measures

#### 2.6.1. Psychosocial Function

We evaluated several indicators of psychosocial function. Social isolation was defined as contact with family or friends less than once a week [36]. Neighborhood relationships were evaluated using a four-point scale [37,38]. Additionally, social participation was measured as the frequency of engagement in community activities such as volunteering, sports groups, hobbies and learning groups [29]. Moreover, psychological aspects include health literacy [39], psychological health [40,41], and depressive mood [42,43].

#### 2.6.2. Physical Activity and Physical Function

Physical activity levels were captured by asking about exercise habits (engaging in exercise at least once a week) and recreational or transport-related walking (150 min or more per week) [44,45]. To assess physical function, we measured frailty status [46,47], higher-level functional capacity as measured using the Tokyo Metropolitan Institute of Gerontology Index of Competence (TMIG-IC) [48], and the Motor Fitness Scale (MFS) [49].

#### 2.6.3. Dietary Habits

We assessed dietary patterns using a dietary variety score and a food frequency score [50,51]. Participants were also asked about the frequency of eating alone [52].

#### 2.6.4. Use of ICT

Participants reported their use of ICT, including the type of device they used at least weekly and their frequency of Internet use for activities such as browsing webpages and exchanging emails [53,54].

### 2.7. Statistical Analyses

After the 2-year follow-up survey, participants were categorized into intervention and control groups based on their participation in any of the CDC project’s interventions. Baseline characteristics are summarized as mean (SD) and frequency (percentage) for continuous and categorical variables, respectively. To reduce bias in background factors between groups, 1:1 propensity score matching was performed using logistic regression based on baseline characteristics, employing nearest neighbor matching with a 0.2 caliper width. To assess the quality of propensity score matching, we calculated the standardized differences for all baseline variables between the intervention and control groups before and after matching. Standardized differences of <0.1 indicate a good balance between groups. The variables included in the propensity score model were age, sex, living arrangement, years of residence in the neighborhood, financial status, employment status, health indicators (self-rated health, musculoskeletal pain, body mass index [BMI]), and lifestyle factors (alcohol and smoking), as these are related factors for older adults to participate in social activities [55,56,57].

Although the initial study protocol specified repeated-measures analysis of variance as the primary analysis [24], we ultimately employed generalized estimating equations (GEE). This change was made to better accommodate the mix of continuous and binary outcomes and to provide more robust handling of the clustered data structure and potential missing values. For each outcome, generalized linear models with GEE were applied to account for within- and between-subject variations. The main effects evaluated were the intervention group, time, and the intervention group × time interaction, assessed using the Wald chi-square test. For continuous outcomes, an identity link with a normal distribution was used, whereas for binary outcomes, a logit link with a binomial distribution was employed. An exchangeable correlation structure with robust standard errors was applied to account for the correlation of the repeated measures.

For sensitivity analyses, we conducted covariate-adjusted GEE analyses, including variables with residual imbalance (standardized mean difference [SMD] > 0.1) after matching [58].

For subgroup analyses, we categorized the intervention participants based on their primary mode of participation (online classes, community hubs, experiential sessions, and smartphone classes) and compared each subgroup with their matched controls using GEE. Notably, our subgroup analyses were exploratory and intended to generate hypotheses for future research. Given the multiple comparisons performed, the risk of Type I error increased, and the findings should be interpreted with caution.

To further elucidate the intervention mechanisms and derive practical implications, we conducted an exploratory subgroup analysis regarding the intervention modalities. Participants in the intervention group were categorized into three mutually exclusive subgroups based on their participation records: (1) “In-person only” (participated only in community hubs or events), (2) “Online only” (participated only in online classes), and (3) “Hybrid” (participated in both in-person and online components). Each subgroup was compared with the matched control group.

A significance level of α = 0.05 indicated statistical significance. Statistical analyses were performed using IBM SPSS Statistics for Windows, version 29.0 (IBM Corp., Armonk, NY, USA) and EZR [59] for calculating SMD.

## 3. Results

### 3.1. Participant Characteristics

**Figure 1:**
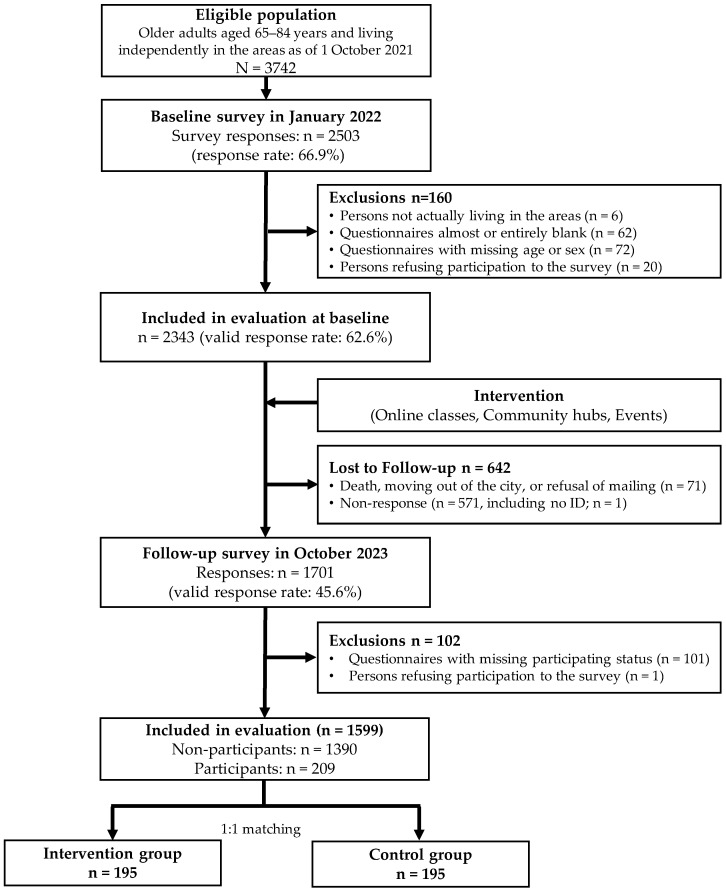
Study Flow Diagram.

Figure 1 illustrates the flow diagram of the study. Of the 3742 eligible individuals who participated in the baseline survey, 2343 provided valid responses (response rate: 62.6%). Overall, 1599 participants completed both surveys after excluding those who died, relocated to other cities, or did not respond to the follow-up survey. Among these, 209 (13.1%) participants reported participating in at least one of the CDC interventions, whereas 1390 were non-participants. After propensity score matching, 195 participants were included in the intervention and control groups each.

Table 1 presents the baseline characteristics of the participants before and after propensity score matching. After propensity score matching, 195 pairs were created, achieving an improved balance in most characteristics (SMD < 0.1), which indicated successful matching. However, some imbalances remained for living alone (SMD = 0.20), years of residence in the neighborhood (SMD = 0.16), and BMI category (SMD = 0.12).

### 3.2. Intervention Effects

The effects of the CDC intervention, estimated using GEE models on the matched sample, are presented in Table 2.

### 3.3. Primary Outcome

No significant difference was observed in the change in SWB between the intervention and control groups over the 2 years (group × time interaction: β = 0.08, 95% confidence interval [CI]: −0.20, 0.37; *p* = 0.565). Mean SWB scores remained stable in both groups, changing from 7.41 (SD 1.82) to 7.45 (SD 1.84) in the intervention group and from 7.50 (SD 1.76) to 7.47 (SD 1.63) in the control group (Table 2).

### 3.4. Secondary Outcomes

As for the secondary outcomes detailed in Table 2, while no significant intervention effects were found for most indicators, including social relationships and physical activity, promising trends were observed in psychological health and health literacy, and a significant improvement was confirmed in ICT use.

Regarding psychosocial outcomes, no significant intervention effects were found for social isolation (*p* = 0.362), neighborhood relationships (*p* = 0.823), or social participation (*p* = 0.586). For psychosocial health, a borderline significant trend suggesting a greater improvement in health literacy (Communicative and Critical Health Literacy [CCHL]) was noted in the intervention group (β = 0.13, 95% CI: 0.00, 0.26; *p* = 0.056). No significant effect was observed for psychological health (World Health Organization-Five Well-Being Index; *p* = 0.358). A non-significant trend toward reduced odds of depressive mood (5-item Geriatric Depression Scale score ≥ 2) was identified in the intervention group (odds ratio [OR] = 0.68, 95% confidence interval [CI]: 0.43, 1.05; *p* = 0.084).

Regarding physical activity and function, no significant intervention effects were found for engagement in exercise (*p* = 0.458), walking habits (*p* = 0.302), frailty status (*p* = 0.858), functional capacity (TMIG-IC; *p* = 0.390), or MFS (*p* = 0.587).

No significant intervention effects were observed for dietary habit outcomes, including the dietary variety score (*p* = 0.743), food frequency score (*p* = 0.186), or eating alone status (*p* = 0.468).

Regarding ICT use, a significant group × time interaction effect was observed for the frequency of Internet use (OR = 1.53, 95% CI: 1.08, 2.16; *p* = 0.016). The proportion of participants using the Internet more than once a day increased from 60.0% to 68.9% in the intervention group and slightly decreased from 51.3% to 50.3% in the control group. No significant effect of smartphone ownership was observed (*p* = 0.211).

### 3.5. Sensitivity Analysis

Sensitivity analyses using GEE with covariate adjustment for residual imbalances (living alone, years of residence, and BMI) yielded results that were largely consistent with those of the primary analysis. The effect on SWB remained non-significant (*p* = 0.439), whereas that on frequent Internet use remained significant (*p* = 0.016). The adjusted model on CCHL had a significant effect on the adjusted model (*p* = 0.044).

### 3.6. Subgroup Analysis

**Figure 2:**
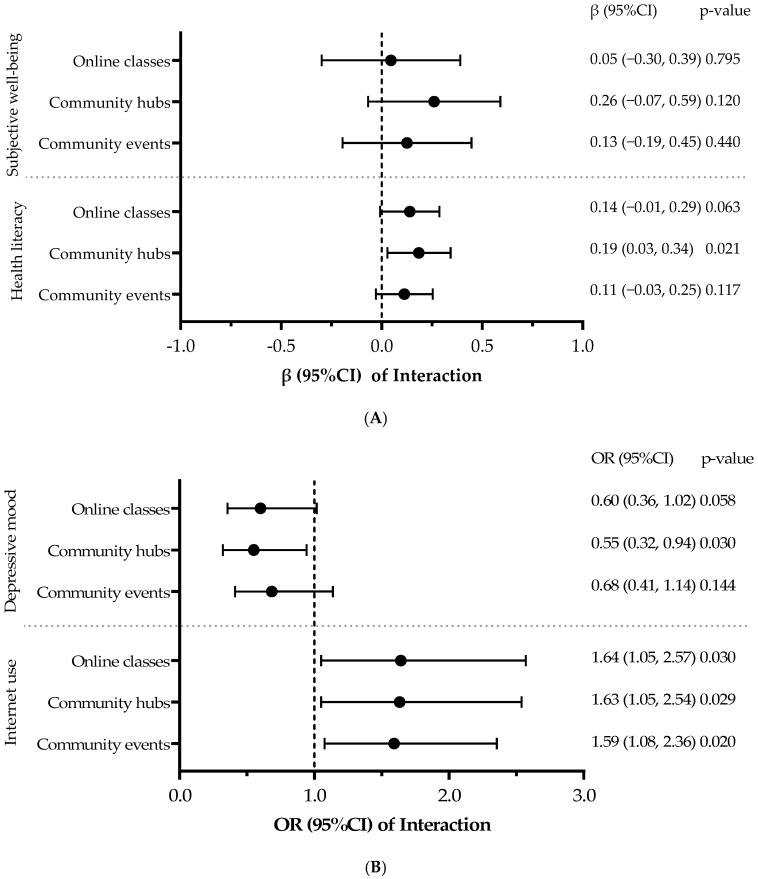
Forest Plots of Subgroup Analyses for Intervention Effects by Participation Type. Note. The figure displays the results of subgroup analyses stratified by the primary mode of participation (online classes, community hubs, community events). The vertical dashed line indicates the null effect (β = 0 or OR = 1) (**A**) The upper two panels show the group × time interaction effects, expressed as unstandardized regression coefficients (β) with 95% confidence intervals (CIs), for continuous outcomes (subjective well-being and health literacy). (**B**) The lower two panels show the interaction effects, expressed as odds ratios (ORs) with 95% CIs, for binary outcomes (depressive mood and frequent Internet use). An OR < 1 indicates reduced odds of depressive mood in the intervention group, while an OR > 1 indicates increased odds of frequent Internet use.

Subgroup analyses stratified based on primary participation type (online classes, *n* = 107; community hubs, *n* = 104; and community events, *n* = 136; these groups were not mutually exclusive and included overlapping participants) revealed no significant improvement in SWB for any subgroup. However, significant increases were observed in frequent Internet use across all subgroups of online classes (*p* = 0.030), community hubs (*p* = 0.029), and community events (*p* = 0.020). Hub visitors showed significant improvements in CCHL (*p* = 0.021) and reductions in depressive mood (*p* = 0.030). As previously described, event attendees demonstrated significant effects, primarily for ICT use outcomes. The results of these subgroup analyses are graphically summarized in Figure 2.

Furthermore, regarding intervention modalities, the “Hybrid” group demonstrated the most robust improvements. Specifically, participants who engaged in both online and in-person activities showed a significant reduction in depressive symptoms (OR = 0.50, 95% CI: 0.28, 0.91; *p* = 0.023) and a significant increase in frequent Internet use (OR = 1.80, 95% CI: 1.07, 3.03; *p* = 0.027). In contrast, single-mode participation (In-person only or Online only) did not yield statistically significant effects compared to controls for these outcomes.

## 4. Discussion

This quasi-experimental study evaluated the effects of a comprehensive community-based intervention combining online and in-person social activities on the SWB of older adults living in an urban Japanese community. Our findings indicate that while the CDC movement intervention did not significantly improve SWB over the 2-year study period, it led to significant improvements in Internet usage frequency and showed promising effects on several secondary outcomes, particularly health literacy and depressive symptoms, within specific participant subgroups.

### 4.1. Primary Outcome: SWB

Contrary to our hypothesis, based on previous meta-analyses suggesting positive effects on well-being [28], we observed no significant improvements in SWB, as measured using the Cantril Ladder. Social relationships, support, and activities are associated with SWB among older adults [6,12,60,61]. However, several factors may explain our findings. First, the intervention may not have been sufficiently intensive or prolonged to affect a relatively stable psychological construct. Although we provided various participation opportunities, the actual engagement dose for most participants may have been insufficient to impact the overall life evaluation. Research has indicated that more intensive, sustained interventions may be necessary to modify social relationships because most interventions are delivered on a weekly or fortnightly basis [62]. Considering the findings from cross-European cohort studies showing that participation in at least one socially productive activity demonstrated a positive correlation with QoL in 2-year follow-ups [63], it is possible that SWB could improve with longer or more intensive intervention exposure. Second, the Cantril Ladder measure, while widely used, may not be adequately sensitive to detect subtle changes in domain-specific aspects of well-being that our intervention may have influenced. The baseline SWB scores in our sample were relatively high (mean of 7.41–7.50 on a 10-point scale) compared with those of other country populations (mean of 6.66) [64], suggesting a potential ceiling effect that limited room for improvement, a phenomenon observed in other well-being interventions. Additionally, a previous review of interventions targeting SWB in low- and middle-income countries reported inconsistent effects [22], indicating that SWB may be challenging to modify across different contexts and populations.

### 4.2. Secondary Outcomes: Digital Engagement and Health Literacy

The most significant and robust achievement of our intervention was the consistent improvement in Internet usage frequency across all participant subgroups. The proportion of participants using the Internet more than once a day increased substantially from 60.0% to 68.9% in the intervention group, whereas it slightly decreased in the control group (51.3% to 50.3%), resulting in a significant intervention effect (OR = 1.53, 95% CI: 1.08, 2.16; *p* = 0.016). This finding represents a critical outcome in an increasingly digitalized society, where technological engagement has become essential for daily functioning, social participation, and accessing health resources.

Digital technology has been shown to enhance self-efficacy among older adults, help maintain connections, and promote physical and mental well-being [65]. Multiple studies have demonstrated that ICT use is positively correlated with happiness and well-being [66]. This digital engagement effect is particularly noteworthy given the persistent “digital divide” affecting older adults globally. A cross-national study revealed that digital exclusion rates vary widely between countries, ranging from 21.1% to 96.9% [67]. Considering that approximately 62.0% of Japanese adults aged 70–75 years use the Internet [68], our intervention successfully bridged this gap using a multifaceted approach that combined targeted smartphone classes, supportive technology-learning environments, and meaningful contexts for digital skill application.

The mechanisms underlying this digital engagement success appear to include both technical instruction and motivational components. The literature suggests that Internet applications can enable individuals to maintain social networks [69], and online social activity can help people maintain their cognitive function [70]. Using the Internet and participating in social activities via ICT may lead to improved SWB, as demonstrated by studies showing associations between Internet use and SWB among older adults [71]. Importantly, recent research has found that Internet use is associated with a reduced probability of depression among older adults [72], and Internet users have a low risk of frailty [73]. These findings suggest that ICT use can improve SWB by mediating enhanced social relationships [14].

Research has consistently demonstrated a significant correlation between health literacy and Internet use among older adults, with lower health literacy being associated with a decreased likelihood of Internet engagement [53,74]. Complementing the digital engagement results, our intervention also demonstrated promising effects on health literacy, with the CCHL scores showing a borderline significant improvement (β = 0.13, 95% CI: 0.00, 0.26; *p* = 0.056) that reached statistical significance in the covariate-adjusted models (*p* = 0.044). This effect was particularly pronounced among community hub participants, suggesting that these social interaction centers created effective environments for developing health literacy skills.

Health literacy improvements are especially significant given the increasingly complex healthcare landscape that older adults must navigate. A review of interventions for older adults’ health literacy found that technology-based interventions were more effective than non-technology-based interventions [75]. Enhanced health literacy enables more effective management of chronic conditions, better healthcare decision-making, more productive healthcare interactions, and ultimately, better health outcomes. Higher levels of health literacy are associated with reduced engagement in health-risk behaviors, including smoking, regular alcohol consumption, and physical inactivity [76,77]. Among older adults, low health literacy contributes to frailty progression [78,79] and mortality [80]. Crucially, both enhanced health literacy and increased digital engagement can empower older adults to foster and maintain online and face-to-face social connections, which could be key determinants of SWB.

### 4.3. Differential Effects by Participation Type

Exploratory subgroup analyses stratified by participation type were conducted to generate hypotheses for future intervention designs. Community hubs appeared to have the broadest impact, showing benefits for health literacy (*p* = 0.021) and depressive moods (*p* = 0.030). This finding suggests that accessible, supportive, and multifunctional community spaces are particularly valuable for promoting holistic health in older populations, serving as “one-stop” settings where multiple needs can be addressed simultaneously. Notably, although the reduction in depressive symptoms among community hub participants (OR = 0.68, *p* = 0.084) did not reach statistical significance in the primary analysis, it supports previous research highlighting the importance of the third place for emotional well-being in later life [33]. A study of social participation patterns found that individuals with depressive symptoms in Japan were more likely to be socially isolated [81]. The emotional support and empathetic listening provided by volunteers at the hubs may have served as protective factors against depression, which may have promoted social activities, even if they did not translate into higher overall SWB.

Online classes demonstrated specific benefits for dietary habits and digital engagement, likely because of the structured nutrition education component, as shown in the intervention [32]. The consistent impact on Internet usage across all participation types suggests that various engagement formats can successfully promote digital inclusion, an important consideration for program planning in diverse communities. This aligns with research showing that ICT can mitigate social isolation in older adults through the following four mechanisms: connecting with the outside world, gaining social support, engaging in activities of interest, and boosting confidence [69].

Our additional analysis revealed that significant improvements in mental health and digital behavior were observed exclusively in the “Hybrid” participation group. This suggests that while the overall intervention did not shift SWB, a synergistic approach combining face-to-face and digital elements may be necessary to foster the psychological changes that precede long-term well-being improvements.

Despite a previous review showing that digital intervention can improve physical activity [82], the positive impact on physical activity was not statistically significant. Nevertheless, Internet users are more likely to meet the recommendations of physical activity than non-users [83].

### 4.4. Strengths, Limitations, and Implementation

This study has several strengths, including its community-based approach, relatively large sample size, comprehensive measurement of outcomes, rigorous analytical methods using propensity score matching and sensitivity analyses, and a relatively long follow-up period of 2 years. The multi-component design enabled the examination of different participation pathways, providing insights into which intervention elements were most effective for specific outcomes.

However, this study had some limitations. First, the quasi-experimental design without randomization is susceptible to selection bias, despite our efforts to minimize this through propensity score matching. The propensity score approach successfully balanced most baseline characteristics; however, some residual imbalances remained, which could have influenced the results despite covariate adjustment in the sensitivity analyses. Although we used propensity score matching to balance observed characteristics, we cannot fully rule out unmeasured differences related to motivation or digital orientation between participants and non-participants. Additionally, the possibility of contamination, whereby individuals in the control group may have been exposed to information about the CDC project through local media or word-of-mouth, cannot be entirely ruled out. Second, participation in the intervention was self-selected and reported, which may have introduced reporting bias and limited causal inference. Previous studies with similar designs have reported higher participation rates, such as 26.1% [84], than those in our study (209/1599 = 13.1%). Third, we could not precisely measure the “dose” of intervention each participant received beyond the type of participation, limiting our ability to conduct dose–response analyses. Future studies would benefit from a more detailed tracking of engagement intensity, frequency, and duration to better understand the relationship between intervention exposure and outcomes. Fourth, the coronavirus disease 2019 pandemic occurred during our study period, potentially affecting both intervention implementation and outcomes. The pandemic necessitated adaptations to program delivery and likely influenced participants’ psychological states, social behaviors, and technology use independently of the intervention. Fifth, our study was conducted in a specific urban Japanese context, which may limit its generalizability to other cultural or geographical settings. Cultural factors, existing social infrastructure, and technological landscapes vary considerably across countries and regions, potentially influencing intervention feasibility and effectiveness.

From a practical perspective, our findings provide a clear direction for future community interventions. The fact that the “Hybrid” group showed the most robust improvements suggests that digital inclusion initiatives should not be implemented in isolation. Instead, they should be integrated with physical community hubs that provide emotional support and a safety net for older adults adopting new technologies. Policy makers should consider designing programs where online skill acquisition is supported by in-person interactions, as this combination appears to be the most effective driver for behavioral and psychological change.

## 5. Conclusions

The CDC intervention, which combined online and in-person activities to promote social connections among older adults, did not significantly improve SWB over 2 years. However, it was successful in increasing Internet usage frequency and showed promising effects on health literacy and depressive symptoms, particularly among specific participant subgroups. These findings indicate that while comprehensive community-based interventions may not immediately shift global evaluations of well-being, they can effectively promote digital inclusion and specific health behaviors among older adults. Future interventions may benefit from more intensive approaches, targeted components for specific outcomes, and consideration of the differential effectiveness of various participation formats.

## Figures and Tables

**Table 1:** Characteristics of Participants in the Original and Matched Cohorts.

	Unmatched Cohort					Matched Cohort				
	Non-Participants		Participants		SMD	Control		Intervention		SMD
	*n* = 1390		*n* = 209			*n* = 195		*n* = 195		
Age, years, *n* (%)										
65–74	737	53.0%	98	46.9%	0.12	97	49.7%	92	47.2%	0.05
75–84	653	47.0%	111	53.1%		98	50.3%	103	52.8%	
Women, *n* (%)	777	55.9%	137	65.6%	0.20	122	62.6%	126	64.6%	0.04
Living alone, *n* (%)	264	19.1%	53	25.4%	0.15	32	16.4%	48	24.6%	0.20
Years of residence in the neighborhood, *n* (%)					0.31					0.16
<29	425	30.6%	43	20.7%		45	23.1%	41	21.0%	
30–59	832	59.9%	154	74.0%		145	74.4%	143	73.3%	
>59	131	9.4%	11	5.3%		5	2.6%	11	5.6%	
Financial status (self-rated), *n* (%)					0.27					0.09
Low	42	3.0%	3	1.4%		2	1.0%	3	1.5%	
Middle-low	183	13.3%	16	7.7%		14	7.2%	16	8.2%	
Middle	535	38.7%	71	34.3%		63	32.3%	65	33.3%	
Middle-high	550	39.8%	102	49.3%		104	53.3%	97	49.7%	
High	71	5.1%	15	7.2%		12	6.2%	14	7.2%	
Employment status (working), *n* (%)	489	35.5%	51	24.8%	0.24	48	24.6%	50	25.6%	0.02
Self-rated health (excellent to good), *n* (%)	1157	83.8%	178	86.0%	0.06	171	87.7%	170	87.2%	0.02
Musculoskeletal pain (either shoulder, low back, or knee), *n* (%)	874	64.6%	136	66.3%	0.04	128	65.6%	129	66.2%	0.01
Body mass index, kg/m^2^, *n* (%)					0.13					0.12
<18.5	102	7.5%	10	4.9%		9	4.6%	10	5.1%	
18.5–24.9	969	71.3%	155	76.0%		158	81.0%	149	76.4%	
≥25	289	21.3%	39	19.1%		28	14.4%	36	18.5%	
Alcohol consumption status, *n* (%)					0.02					0.03
Never or former	602	43.4%	93	44.5%		84	43.1%	87	44.6%	
Current	785	56.6%	116	55.5%		111	56.9%	108	55.4%	
Smoking status, *n* (%)					0.28					0.07
Never or former	1265	91.3%	204	97.6%		192	98.5%	190	97.4%	
Current	120	8.7%	5	2.4%		3	1.5%	5	2.6%	

Note. Values are presented as the means ± standard deviations and total numbers (percentages) for continuous and categorical variables, respectively. SMD, standardized mean difference.

**Table 2:** Effects of the CDC Intervention on Primary and Secondary Outcomes.

						Intervention Effects (Group × Time)			
		Baseline		Follow-Up			95% CI		*p*-Value
Subjective well-being, mean (SD)	Control	7.50	1.76	7.47	1.63	β = 0.08	−0.20	0.37	0.565
	Intervention	7.41	1.82	7.45	1.84				
Social relationships									
Social isolation, *n* (%)	Control	49	26.1%	63	33.7%	OR = 1.25	0.78	2.00	0.362
	Intervention	35	18.8%	54	28.9%				
Neighborhood relationships, *n* (%)	Control	123	66.1%	129	66.8%	OR = 1.05	0.67	1.65	0.823
	Intervention	136	73.5%	140	74.9%				
Social participation more than once a month, *n* (%)	Control	88	45.6%	87	44.8%	OR = 1.11	0.77	1.59	0.586
	Intervention	134	69.1%	137	70.6%				
Health literacy (CCHL), mean (SD)	Control	3.71	0.79	3.64	0.70	β = 0.13	0.00	0.26	0.056
	Intervention	3.70	0.74	3.77	0.68				
Psychological health (WHO-5: 0–25), mean (SD)	Control	15.57	5.24	15.32	5.16	β = 0.41	−0.46	1.28	0.358
	Intervention	15.83	5.30	15.87	5.11				
Depressive mood (GDS-5 ≥ 2), *n* (%)	Control	53	28.2%	68	36.2%	OR = 0.68	0.43	1.05	0.084
	Intervention	67	35.6%	65	34.8%				
Physical activity and physical function									
Engaging in any exercise more than once a week, *n* (%)	Control	156	83.4%	154	81.5%	OR = 1.26	0.68	2.35	0.458
	Intervention	163	87.2%	166	87.8%				
Engaging in walking ≥150 min per week, *n* (%)	Control	141	86.5%	130	85.0%	OR = 1.43	0.73	2.82	0.302
	Intervention	136	81.0%	139	85.8%				
Frailty (CL15 score ≥ 4), *n* (%)	Control	29	15.8%	26	14.4%	OR = 1.06	0.59	1.89	0.858
	Intervention	32	17.5%	32	17.2%				
TMIG-IC (score: 0–13), mean (SD)	Control	11.70	1.55	11.47	1.76	β = 0.11	−0.14	0.36	0.390
	Intervention	11.58	1.60	11.43	1.62				
Motor Fitness Scale score, mean (SD)	Control	10.66	3.43	10.08	3.69	β = 0.13	−0.35	0.61	0.587
	Intervention	10.87	3.09	10.43	3.34				
Dietary variety									
Dietary variety score (0–10), mean (SD)	Control	3.69	2.25	3.78	2.36	β = 0.06	−0.32	0.44	0.743
	Intervention	3.88	2.31	4.05	2.19				
Food frequency score (0–30), mean (SD)	Control	18.96	5.09	19.08	5.25	β = 0.46	−0.22	1.14	0.186
	Intervention	19.49	4.91	20.10	4.55				
Eating alone at least a whole day per week, *n* (%)	Control	79	42.5%	75	41.9%	OR = 1.15	0.79	1.66	0.468
	Intervention	98	52.1%	99	54.1%				
Use of ICT									
Owning a smartphone, *n* (%)	Control	129	66.5%	133	69.3%	OR = 1.37	0.84	2.23	0.211
	Intervention	158	81.4%	170	87.2%				
Using the Internet more than once a day, *n* (%)	Control	98	51.3%	95	50.3%	OR = 1.53	1.08	2.16	0.016
	Intervention	114	60.0%	131	68.9%				

Note. SD, standard deviation; CI, confidence interval; CCHL, Communicative and Critical Health Literacy; WHO-5, World Health Organization-Five Well-Being Index; GDS-5, 5-item Geriatric Depression Scale; CL15, Check-List 15 (frailty); TMIG-IC, Tokyo Metropolitan Institute of Gerontology Index of Competence; ICT, information and communication technology; OR, odds ratio; CDC, Chofu–Digital–Choju.

## Data Availability

The data presented in this study are available on request from the corresponding author due to ethical restrictions.

## Supplementary Materials

The following supporting information can be downloaded at: https://www.mdpi.com/article/10.3390/geriatrics11020027/s1, Table S1: TREND Statement Checklist.

## Abbreviations

BMI	Body mass index
CCHL	Communicative and Critical Health Literacy
GEE	Generalized estimating equations
ICT	Information and communication technology
QoL	Quality of life
SD	Standard deviation
SWB	Subjective well-being
